# Speech intelligibility and recall of first and second language words heard at different signal-to-noise ratios

**DOI:** 10.3389/fpsyg.2015.01390

**Published:** 2015-09-14

**Authors:** Staffan Hygge, Anders Kjellberg, Anatole Nöstl

**Affiliations:** Environmental Psychology, University of GävleGävle, Sweden

**Keywords:** noise, recall, speech intelligibility, word lists, signal-to-noise ratio, working memory, working memory capacity

## Abstract

Free recall of spoken words in Swedish (native tongue) and English were assessed in two signal-to-noise ratio (SNR) conditions (+3 and +12 dB), with and without half of the heard words being repeated back orally directly after presentation [shadowing, speech intelligibility (SI)]. A total of 24 word lists with 12 words each were presented in English and in Swedish to Swedish speaking college students. Pre-experimental measures of working memory capacity (operation span, OSPAN) were taken. A basic hypothesis was that the recall of the words would be impaired when the encoding of the words required more processing resources, thereby depleting working memory resources. This would be the case when the SNR was low or when the language was English. A low SNR was also expected to impair SI, but we wanted to compare the sizes of the SNR-effects on SI and recall. A low score on working memory capacity was expected to further add to the negative effects of SNR and language on both SI and recall. The results indicated that SNR had strong effects on both SI and recall, but also that the effect size was larger for recall than for SI. Language had a main effect on recall, but not on SI. The shadowing procedure had different effects on recall of the early and late parts of the word lists. Working memory capacity was unimportant for the effect on SI and recall. Thus, recall appear to be a more sensitive indicator than SI for the acoustics of learning, which has implications for building codes and recommendations concerning classrooms and other workplaces, where both hearing and learning is important.

## Introduction

When the teacher’s speech signal is degraded by the acoustic properties of the classroom, speech intelligibility is reduced, which in turn makes learning more difficult. In order to minimize acoustic disturbances in the classroom, government agencies have established building codes, standards, and recommendations for acceptable signal-to-noise ratios (SNRs) and reverberation time in classrooms and other work places, where it is important to hear and understand auditory information (American National Standards Institute, 2002; Vallet and Karabiber, 2002; Swedish Work Environment Authority, 2006, 2011; Swedish Standards Institute, 2007). These codes and standards are based on what is required for correct identification of spoken words or isolated sentences, i.e., speech intelligibility (SI), which mostly is defined as percentage or probability of correct identifications.

However, SI or correct identification of the spoken word is only one factor in memorizing the information and probably not enough. Acceptable listening conditions are no guarantee for good learning. Kjellberg (2004) argued that if the acoustic conditions or other factors make listening harder or requiring more effort, the recall will suffer even if SI is at an acceptable level. The key factor for the impaired recall seems to be that when the limited working memory capacity is depleted, less time and resources are left for processing and storing of the material to be remembered. In two experiments, Kjellberg et al. (2008) and Ljung and Kjellberg (2009) found support for this hypothesis. Similar results have also been reported in other recent papers from our group (Ljung et al., 2009, 2013), as well as by others in earlier studies (Rabbitt, 1966, 1968; Surprenant, 1999, 2007).

One implication of these results is that SI may be a cruder indicator of the quality of the listening conditions than the memory and recall of the spoken message. In order to show that, SI and recall should be assessed independently for the same material and by the same subjects. In earlier studies from our group (Kjellberg et al., 2008; Ljung and Kjellberg, 2009) the participants shadowed all the words they heard in the wordlist. This was done to ensure that the words were captured correctly also in the less favorable listening conditions.

Previous research also indicates that shadowing suppresses the free recall of the early items of on a word list (Petrusic and Jamieson, 1978; cf. also Parkinson et al., 1971; Parkinson, 1972). In our context it was further important to explore whether shadowing influenced the effect on recall also in an unfavorable listening conditioning, such as +3 dB SNR, and whether recall may be an advantage of recall for late items in a wordlist, resulting in over- or underestimation of recall.

A related issue is whether SNR has a more pronounced effect on the memory of second language words than on the native tongue words, even if the SI is equal. This would be expected if encoding of the second language words requires more processing resources, i.e., will be more taxing for working memory.

SNR also interacts with the position of the word in a wordlist. Kjellberg et al. (2008) found that the free recall decreased in the primacy and recency parts of spoken word lists when the words were presented at a lower SNR. As primacy and recency effects are assumed to reflect long- and short-term memory respectively, we wanted to further explore the extent to which SNR had different effects for long- and short-term memory.

The present experiment was designed to investigate these questions. For the recall task four variables were selected as within person factors: (i) whether the spoken wordlists were in Swedish (native tongue) or in English (Language), (ii) whether the words were heard under acceptable or less than acceptable SNR (+12 or +3 dB), (iii) whether the spoken words were shadowed orally directly after presentation or not (Shadowing), and (iv) whether the presented word was in the first, second or third part of the word list (Part). Thus, all participants encountered all experimental combinations of Language, SNR, Shadowing, and Part. In addition, the outcome of a pre-experimental working memory operation span (OSPAN) task was split by the median and included as an between person independent variable of working memory capacity. In a previous study (Ljung et al., 2013), OSPAN was reported to be related to recall, but not to SI.

For the SI, SI task, the probability of correctly identified words in the shadowing task, was analyzed with Language, SNR, and OSPAN as independent variables. In the SI task, the factor Part was not meaningful as the participants repeated back each word immediately after hearing it.

For SI we expected main effects of SNR and Language, but conjectured that the size of the SNR-effect would be higher for recall than for SI.

For the recall of the words the basic hypotheses were that for the +3 dB compared to +12 dB SNR, recall would be worse, which would also be the case for English words compared to the Swedish words. The size of the loss in recall from SNR for English words was expected to be larger than for Swedish words. Our OSPAN measure of working memory capacity was expected to show up both as a main effect and in interactions with SNR or Language.

## Materials and Methods

### Participants

A sample of 48 undergraduate students with a mean age of 27.1 years (SD = 7.8) and with equal numbers of men and women participated in the study. They were recruited by information screens in the university premises. Self-reported normal hearing, reading and writing skills were inclusion criteria and the subjects received a cinema ticket for their participation. All participants had studied English for 9 or 10 years before they entered university studies, at which level most readings for their courses are in English. Thus, their English proficiency is quite high. None of the participants had taken English at university level. On arrival the participants were informed about the study, and about their right to leave the experiment at any time without giving any reason. On a direct question all the subjects agreed to participate. For this research we have an ethical approval from the Regional Ethical Board in Uppsala (Nr 338/2011), which allows to take an informed verbal consent, rather than a written one, given that it is documented by whom, to whom, where and when the consent was given. This was done in the present study.

### Word Lists

Twenty-four word lists with twelve words each were generated, twelve lists in English and 12 in Swedish. The words were taken from 24 semantic categories and chosen from category norms for the two languages in which the words are ranked with respect to the strength of their association with the category. For the Swedish words category norms reported by Nilsson (1973) and Hellerstedt et al. (2012) were consulted. For the English words we relied on works by Battig and Montague (1969), Posnansky (1978), Marshall and Parr (1996) and van Overschelde et al. (2004).

Because the subjects were native Swedish speakers, the English word lists were slightly modified to reduce any SI difference between the Swedish and English lists. A few English words which were judged by the first author to be uncommon to the participants were replaced with more common ones. The average number of syllables were about the same for the English and Swedish words (*F* < 1, means English: 1.62, Swedish: 1.65) and there was no significant interaction between Language and Part of the word lists in this respect. Thus, it can be said that the difference in difficulty between the English and Swedish words lists were not a mere reflection of the length of the words and the number of syllables.

The average category norm rank orders of the individual words were made equal for all the lists (Graeco-Latin squares). Three sets with eight lists each and with counterbalanced presentation orders in the eight combinations of language, SNR, and shadowing were generated. The words were recorded in one session from a female speaker, fluent in both English and Swedish, in a sound-attenuated chamber and normalized to 66 dB(A). The words were read to the participants with a 3 s interval between the words. Broad band noise was added to the word lists to create the SNR conditions of +12 and +3 dB. The lists were presented to participants via Sennheiser HD-202 headphones. All the equipment, including computers (Dell) that the participants used was of the same make and model.

Participants were instructed to memorize as many of the spoken words as possible. After each list participants were given 1 min to type down the words they could remember from the most recent list. The computer model was the same for all participants. This procedure continued until all 24 lists had been presented. The probability for recall of the presented words was the basis dependent measure, and the participants were given a score of 1 for each correctly recalled word even if the spelling was not perfect.

For the half of the word lists that made up the SI shadowing task, 12 in each language, the participants were instructed to repeat aloud the words they heard (shadowing). The lists where shadowing occurred were counterbalanced for the presentation orders in the crossed combinations of SNR and Language. The participants’ verbal responses were tape recorded and they were given 0 or 1 as probability scores for the 12 words in each word list, even if the pronunciation was not perfect.

### Operation Span

A Swedish translation of the automated OSPAN task (Unsworth et al., 2005) was administered as a pre-experimental measure of working memory capacity. Mathematical operations (e.g., “Is (5 + 3) × 3 = 24?”) were presented on a computer screen. The participant was told to respond “yes” or “no” to the operation, as quickly as possible, by pressing a button on the screen using the computer mouse. When a response was recorded, a letter was presented for 0.8 s and the participant was told to remember it for later recall. After that a new mathematical operation was presented or the list ended. The list lengths varied between 3 and 7 letters. A total of 15 lists were used (3 of each list length), and the length increased across the task. When a list ended, the participants were asked to recall the letters in order of presentation. Points were given for each word recalled in the correct serial position and the score for each list was multiplied by the length of the list in order to balance differences in list difficulty. The accumulated points were divided by the total amount of lists (i.e., 15), yielding a maximum possible score of 27 (the maximum observed score was 26.5).

### Procedure

Between one and three participants were tested in each session. Each participant was seated with headsets on in front of an individual laptop in a sound attenuated test-room. All participants started with the self-paced OSPAN task.

After the OSPAN task the participants adjusted the listening level in the headphones to a comfortable level, and began with a training phase in which they listened to two lists each from the two languages, with the two SNR levels crossed with the two levels of shadowing. After the training phase the 24 wordlists were presented. The duration of each word was approximately 1 s with a 3-s interstimulus interval. The presentation order of the lists was pseudo-randomized and counter balanced for each set of eight participants. The window for typing in the recalled words remained open for 60 s and was followed by the playback of the next list. The total session lasted 55–65 min depending on how fast participants completed the OSPAN task.

### Statistical Analyses

The OSPAN scores were split by the median to form one group with high OSPAN-scores and one group with low scores (Means: High – 22.05, Low – 15.20; *SD*: High – 2.45, Low – 2.65).

For the analysis of SI-shadowing a split-plot ANOVA was performed with Language and SNR as within-subject factors and OSPAN as a between-subject factor. For the analysis of the recall scores Shadowing and the three Parts of the wordlists were added on as within-person variables. That is, position 1–4 in the list were defined as Part 1, position 5–8 as Part 2, and position 9–12 as Part 3.

Thus, separate ANOVAs were run for SI and recall, not a grand MANOVA for them together because we had SI scores for only half of the lists and also that the variable Part did not make sense in the immediate response asked for in the SI-task.

## Results

When reporting the results, decimals in the degrees of freedom for the *F*-tests indicate that a Greenhouse–Geisser correction was made because of violations of the sphericity assumption.

### SI-Shadowing

In the SI shadowing task, three participants (two males, one female) were excluded because of recording errors or for not following instructions. There was no main effect of OSPAN on SI [*F*(1,43) = 0.482, *p* > 0.10], and no significant interactions between OSPAN and the other independent variables or their combinations (all *p*s > 0.10) and, therefore, the subsequent SI-analyses were performed without the OSPAN dichotomization, and with Language and SNR as the independent variables. The was a significant main effect of SNR [*F*(1,44) = 11.63, *p* < 0.001, Cohens *d* = 0.50, Means: +3 dB = 11.13, +12 dB = 11.60] indicating more of the 12 words in each list was correctly shadowed with the higher dB-value. There was no significant main effect of Language [*F*(1,44) = 2.26, *p* > 0.10], and there was no significant interaction SNR × Language. Thus, for SI there was only a marked main effect of SNR with a medium effect size. The lack of any effects of Language strongly indicates that the Swedish and English lists did not differ in SI.

### Free Recall

Also for the free recall task there was no main effect of OSPAN [*F*(1,46) = 1.05, *p* > 0.10; **Table 1**]. An inspection of all the interactions between OSPAN and all the other four independent variables in all 15 combinations only yielded one single significant interaction, Language × Part × OSPAN (*p* = 0.046), which was deemed to be of minor importance and being too close to what 5% pure chance mass significance would yield. Thus, to increase group size, power, reliability and sensitivity the subsequent analyses of recall were made without the OSPAN factor, leaving Language, Shadowing, SNR and Part as the independent variables for the free recall task.

**Table 1:** Means and *F*-ratios for the main effects on recall.

Variance source	Recall, means probability		*F*-test – Greenhouse–Geisser *df-* adjusted for the source *Part*	Cohen *d* for some of the effects
Language	English: 0.423	Swedish: 0.461	*F*(1,47) = 24.743, *p* < 0.001	0.72
Shadowing	No: 0.454	Yes: 0.430	*F*(1,47) = 6.788, *p* = 0.012.	
OSPAN	Low: 0.420	High: 0.464	*F*(1,46) = 1.050, *p* = n.s.	
Signal-to-noise ratio (SNR)	+3 dB: 0.418	+12 dB: 0.466	*F*(1,47) = 49.403, *p* < 0.001	1.01
Part	1: 0.394 2: 0.342 3: 0.590		*F*(1.29,60.58) = 69.048, *p* < 0.001	

For the free recall task the main effects are shown in **Table 1**. Note the high Cohen *d* for SNR (1.01), which is noticeably higher than for the SI-shadowing task above (*d* = 0.50), and the close to strong effect of Language (0.72).

**FIGURE 1:**
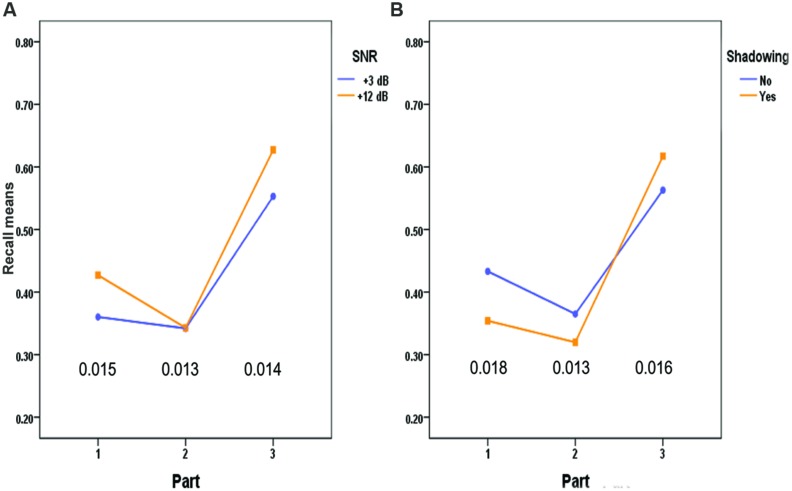
**Recall of words in the three Parts of the word lists by SNR (A) and Shadowing (B).** The values at the bottom of the figures are the standard errors of the mean differences between the vertically oriented pairs of means.

**FIGURE 2:**
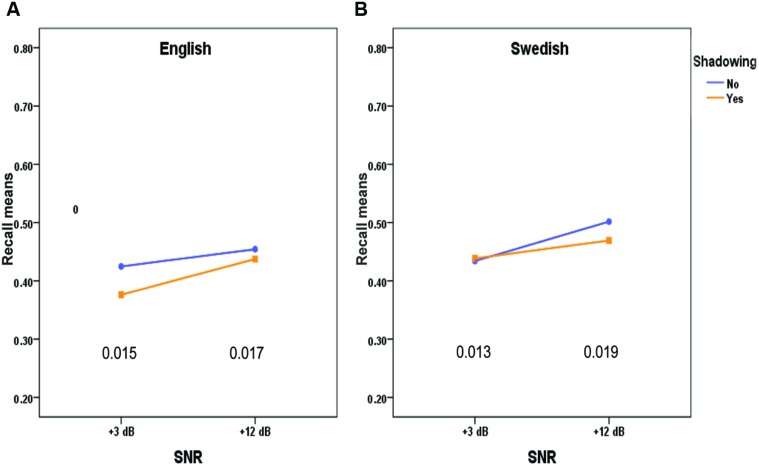
**Recall of words in English (A) and Swedish (B) by SNR and Shadowing.** The values at the bottom of the figures are the standard errors of the mean differences between the vertically oriented pairs of means.

**Table 2** and **Figures 1** and **2** show the resulting significant interactions between our experimental variables on recall.

**Table 2:** *F*-ratios for the significant interactions of the independent variables on free recall.

Variance source	*F*-test – Greenhouse–Geisser *df*-adjusted for the sources including *Part*	Power	$\eta_{p}^{2}$
Shadow × part	*F*(1.81,84.90) = 18.29, *p* = 0.000	1.00	0.280
SNR × part	*F*(1.99,93.63) = 7.49, *p* = 0.001	0.94	0.140
Language × shadow × SNR	*F*(1,47) = 5.07, *p* = 0.029	0.60	0.097

As seen from the general form of the curves in **Figure 1**, recall is best at the end of the word list (recency effect), and second best at the beginning of the list (primacy effect). This reflects the well-known serial position effect. **Figure 1** also shows the significant interactions SNR × Part, and Shadow × Part, and the numerical details of these interactions are given in **Table 2**

**Figure 1A** indicates that that the higher SNR makes a positive difference at the beginning and at the end of the word lists, but not in the middle of the wordlist. A test of simple main effects of SNR in the three Parts of the word lists revealed significant effects of SNR in Part 1 (*p* < 0.000) and Part 3 (*p* < 0.000), but not in Part 2 (*p* = 0.947). That is, the higher (+12 dB) SNR value was an advantage in the first and last parts of the lists, but not in the middle part.

**Figure 1B** shows the shifting advantage from shadowing the words. In the first two parts shadowing *impaired* recall of the words, but in the last part there was an advantage of having repeated the words. A test of simple main effects of Shadowing in the three parts of the word list showed significant effects (all *p*s < 0.005) for all three pairwise comparisons, but the direction of the differences changed in the third part of the list. Thus, shadowing the words interfered with, rather than enhanced the subsequent recall of the words in the first two-thirds of the list.

**Figure 2** shows the significant three-way interaction Language × Shadowing × SNR. For the English words lists, there was a significant simple main effect of shadowing at SNR +3 dB (*p* < 0.005), but not at SNR +12 dB (*p* = 0.318). For the Swedish word lists there were no significant simple main effect of shadowing neither at SNR +3 dB (*p* < 0.723), nor at SNR +12 dB (*p* = 0.088). Thus, shadowing seems to be a more important negative variable for the recall of English word lists, than for the native tongue Swedish word lists, in its effect on recall at +3 dB.

In summary, the main findings were that both SI and recall was impaired in the unfavorable listening condition (+3 dB), but the effect size was larger for recall than for SI. Language also had a main effect on recall, with a medium effect size, but Language did not have any significant effect on SI. Further, the effect of shadowing on recall was negative for the first two parts of the list, but positive for the last part. Shadowing had no general effect on the effect of SNR on recall, but for the English word lists it added to the negative effect in the +3 dB condition.

## Discussion

A notable feature in the results is the difference between the performance on the SI in the shadowing task and the free recall of the words. For the variables that were the same across the two tasks, SNR had a strong main effect for both SI and recall, but the effect size for the effect on recall was higher (1.01) than for the effect on SI (0.50). For language there was a marked effect on recall with an effect size of 0.72, which approached a strong effect, but language did not have any significant effect on SI. As there was no difference in SI between the Swedish and English wordlists, the effects reported on recall are not a matter of the participants not having heard the English words as good as the Swedish words. An explanation of the effects on recall then must be sought elsewhere, and our suggestion is centered on the limited capacity of the working memory, which makes it harder to elaborate, analyze and memorize the English words, even if they are as intelligible as the Swedish words.

The results support our basic hypothesis, that recall is a more sensitive indicator than SI when assessing the acceptability of the acoustic conditions in premises, like schools where understanding and memory of spoken information is critical. Thus, it would be more relevant to base acoustic norms and recommendations on memory and recall rather than on SI.

For the recall task, the effects varied between the three parts of the wordlists. The positive effect of the +12 vs. +3 dB SNR was seen both in the first and last part, but not in the middle part. One interpretation of this can be based on what is thought about the nature of the serial recall learning curve, where the early parts of the curve are seen as a consequence of more opportunities for rehearsal, and thereby a more efficient transfer into the long-term memory. The more words that are added to the list, the less are the possibilities to rehearse all preceding words, leading to a less efficient transfer to long term memory. Recall of the last part of the list is assumed to reflect short-term memory. Along this argument it can be argued that the words heard at +3 dB need more working memory resources than the +12 dB words, and thus less capacity is left for storing and retrieval at SNR +3 dB.

A somewhat surprising effect of shadowing was that it had a positive effect on recall only at the end of the lists. The negative effect in the first and second parts is consistent with previous research (Parkinson et al., 1971; Parkinson, 1972; Petrusic and Jamieson, 1978) and seen as an overall negative net effect of shadowing. Shadowing in the first two parts of the wordlists probably impaired recall by interfering with rehearsal of the preceding words. Rehearsal of the words in the last part of the list in memory seem to be less important for recall because they are within the time reach of the echoic memory (there was about 12 s from the first word in Part 3 of the list until typing in the recalled words). Therefore, the elaboration and rehearsal of the words required when shadowing might have had a positive effect on recall.

Shadowing did not have a general effect in the unfavorable listening condition (+3 dB) but the interaction Language × Shadowing × SNR, as depicted in **Figure 2**, suggests that it has such an effect when the list contains second language words. One explanation of this effect is that some of the words in English, which were not more difficult to shadow, still took more of working memory resources than Swedish words at the low SNR-level, which then resulted in inferior recall.

Contrary to the hypothesis, the more unfavorable listening condition did not have a more marked detrimental effect on the memory of the English lists compared to the Swedish ones, indicated by the lack of an interaction Language × SNR. A possible explanation is that the English words were so well-known to the student participants that they were as easily identified as the Swedish ones. The recall of the last words (position 12) in each wordlist under shadowing and at +12 dB SNR did not reveal any significant difference in recall between English and Swedish words (Means 0.85 and 0.88, *F* < 1, and this non-significant difference was true for all the three blocks of list presentations, all pairwise *F*s < 1.63). Thus, with the lists used in this study the two languages might have been at approximately the same comprehension level.

From an applied perspective it would have been an advantage to have an estimate of how difficult the English word list were in comparison with the Swedish lists for the group we studied. However, from a basic experimental point of view and in the analysis of variance it is quite admissible to compare levels of independent variables, such as difficulty of English and Swedish words, even if we do not have a magnitude measure of the degree of difference between the two levels.

It can also be argued that the category norms for the English words in our words lists should have been assessed in sample similar to the one we used to avoid the problem that the “true” category norm count for the English words when presented to our Swedish college students may not be the same as for first language English speakers. However, we decided not to do that, because that would a too large project of its own, but in a way we came fairly close to having comparable probabilities between the English and Swedish words as there was no significant effect of Language for the SI-shadowing task. (See Results – SI-shadowing).

From the ecological relevance point of view, the learning of word lists is a rare task outside the laboratory. However, similar effects to those reported here have been shown for memory of lectures listened to in different acoustic conditions (Ljung et al., 2009). A better and ecologically more valid test of the effect of language would probably be to study memory for a text in English and Swedish. In such a situation, it is likely that the interpretation of the meaning of the English text would require more working memory resources, and the difference in recall between the two languages would be more pronounced.

Further studies are wanted to use these results for more direct acoustic recommendations for learning. As of now we can only conclude that recall and memory seem to be a better and more sensitive indicator than SI of the acoustic conditions. However, we do not know the exact range of the SNR to produce decrements in recall. It may well be the case that also a SNR of +12 dB is not the best SNR for good recall.

In a forthcoming study we will have more to say about acoustic conditions and recall of word lists, and whether the introduction of two levels of reverberation times interacts, or not, with the same SNR-levels as used in the present study. Doing that will give more empirical facts in the process of re-evaluating building codes and recommendation for the acoustic conditions in rooms, such as class rooms, where not only listening, but also memory and learning are important.

## Conflict of Interest Statement

The authors declare that the research was conducted in the absence of any commercial or financial relationships that could be construed as a potential conflict of interest.
